# PANDA: a pipeline toolbox for analyzing brain diffusion images

**DOI:** 10.3389/fnhum.2013.00042

**Published:** 2013-02-21

**Authors:** Zaixu Cui, Suyu Zhong, Pengfei Xu, Yong He, Gaolang Gong

**Affiliations:** State Key Laboratory of Cognitive Neuroscience and Learning, Beijing Normal UniversityBeijing, China

**Keywords:** PANDA, diffusion MRI, DTI, pipeline, diffusion metrics, structural connectivity, network, connectome

## Abstract

Diffusion magnetic resonance imaging (dMRI) is widely used in both scientific research and clinical practice in *in-vivo* studies of the human brain. While a number of post-processing packages have been developed, fully automated processing of dMRI datasets remains challenging. Here, we developed a MATLAB toolbox named “Pipeline for Analyzing braiN Diffusion imAges” (PANDA) for fully automated processing of brain diffusion images. The processing modules of a few established packages, including FMRIB Software Library (FSL), Pipeline System for Octave and Matlab (PSOM), Diffusion Toolkit and MRIcron, were employed in PANDA. Using any number of raw dMRI datasets from different subjects, in either DICOM or NIfTI format, PANDA can automatically perform a series of steps to process DICOM/NIfTI to diffusion metrics [e.g., fractional anisotropy (FA) and mean diffusivity (MD)] that are ready for statistical analysis at the voxel-level, the atlas-level and the Tract-Based Spatial Statistics (TBSS)-level and can finish the construction of anatomical brain networks for all subjects. In particular, PANDA can process different subjects in parallel, using multiple cores either in a single computer or in a distributed computing environment, thus greatly reducing the time cost when dealing with a large number of datasets. In addition, PANDA has a friendly graphical user interface (GUI), allowing the user to be interactive and to adjust the input/output settings, as well as the processing parameters. As an open-source package, PANDA is freely available at http://www.nitrc.org/projects/panda/. This novel toolbox is expected to substantially simplify the image processing of dMRI datasets and facilitate human structural connectome studies.

## Introduction

Diffusion magnetic resonance imaging (dMRI) has become one of the most popular MRI techniques for brain research. dMRI can be used to quantify white matter (WM) property and to virtually reconstruct WM pathways in the living brain (Le Bihan, 2003). Given its unique merits, dMRI has been extensively applied to the study of WM connectivity in both normal and abnormal conditions, leading to a substantial enhancement in our understanding of the role of WM, particularly in brain diseases (Johansen-Berg and Rushworth, 2009).

One popular application of dMRI is to extract various diffusion metrics [e.g., fractional anisotropy (FA) and mean diffusivity (MD)] that putatively reflect WM integrity (Basser and Pierpaoli, 1996; Pierpaoli and Basser, 1996; Beaulieu, 2002). These metrics can be further applied to identify differences in WM integrity across subjects. To perform this type of analysis, multiple sequential image-processing steps (e.g., eddy-current correction, tensor calculation, metric calculation, and normalization) are required. Currently, a number of packages, such as FMRIB Software Library (FSL) (Smith et al., 2004) and DTI-Studio (Jiang et al., 2006), provide a set of functions that can carry out these jobs. However, these packages typically perform the processing step-by-step and subject-by-subject. Obviously, this processing pattern is inefficient, as users have to wait until the preceding steps or until each subject is completely finished before initiating the next step or subject. In addition, this pattern requires a large amount of manual operation, which potentially increases the possibility of processing errors caused by manual mistakes. To date, a toolbox supporting fully automated processing of raw dMRI datasets to diffusion metrics that are ready for statistical analysis is still lacking.

Another popular application of dMRI is to virtually reconstruct WM tracts, referred to as diffusion tractography (Mori et al., 1999; Behrens et al., 2007). Previous studies using diffusion tractography mainly focus on a few specific WM tracts. Recently, accurately constructed entire brain anatomical networks (i.e., the connectome) based on diffusion tractography have attracted a lot of attention (Behrens and Sporns, 2012) and are the key target of the ongoing human connectome project (http://humanconnectome.org/). While the framework for constructing anatomical networks of the human brain (i.e., definition of network nodes and edges) has been established (Hagmann et al., 2008; Gong et al., 2009a,b), it is mainly implemented in-house. The community is in urgent need of a fully automated public tool that can construct anatomical brain networks using dMRI datasets.

Currently, there have been a few packages such as MIPAV (McAuliffe et al., 2001), JIST (Lucas et al., 2010), Nipype (Gorgolewski et al., 2011), and LONI (Dinov et al., 2009), which aim to facilitate automated processing of neuroimaging dataset. Essentially, these packages provide environments for constructing analysis workflows with a number of pre-included processing modules from existing tools (e.g., Camino, FSL, AFNI, FreeSurfer, and SPM), and therefore various automated processing pipelines (e.g., a dMRI processing pipeline) can be developed within these environments. In order to construct pipelines with these packages, users need to choose processing modules and define dependencies and parameters themselves. It is noted that, if particular processing modules are not encapsulated [e.g., JIST does not include Tract-Based Spatial Statistics (TBSS) analysis], users have to develop their own modules and further incorporate them into the environment. While these powerful and sophisticated packages make it possible to generate a dMRI processing pipeline, they are favored by developers, and not end users without programming skills. A ready-for-use pipeline tool for dMRI processing is highly desired, particularly for end users.

Here, we present a MATLAB toolbox named PANDA (a Pipeline for Analyzing braiN Diffusion imAges) for a comprehensive pipeline processing of dMRI dataset, aiming to facilitate image processing for the across-subject analysis of diffusion metrics and brain network constructions. Of note, the processing pipelines in this toolbox have been completely set up, allowing the end-users of dMRI to process the data immediately. Moreover, the processing procedures within this pipeline were carefully designed to follow the recommended practice as possible (Jones et al., 2012). After the user sets the input/output and processing parameters through the friendly graphical user interface (GUI), PANDA fully automates all processing steps for datasets of any number of subjects, and results in data pertaining to many diffusion metrics that are ready for statistical analysis at three levels (Voxel-level, ROI-level, and TBSS-level). Additionally, anatomical brain networks can be automatically generated using either deterministic or probabilistic tractography techniques. Particularly, PANDA can run processing jobs in parallel with multiple cores either in a single computer or within a distributed computing environment using a Sun Grid Engine (SGE) system, thus allowing for maximum usage of the available computing resources.

To assess the usability and validity of PANDA, we apply PANDA to study the age effect (i.e., old vs. young) on the diffusion metrics of WM as well as the topological properties of the WM network. According to previous findings, decreased WM anisotropy and weakened network efficiency are expected in old individuals.

## Materials and methods

PANDA was developed by using MATLAB under an Ubuntu Operating System. A number of processing functions from FSL (Smith et al., 2004), Pipeline System for Octave and Matlab (PSOM) (Bellec et al., 2012), Diffusion Toolkit (Wang et al., 2007), and MRIcron (http://www.mccauslandcenter.sc.edu/mricro/mricron/) were called by PANDA. Here, we will describe the procedures of pipeline processing in PANDA, followed by an introduction to the realization of pipelines.

### PANDA processing procedures

**Figure 1:**
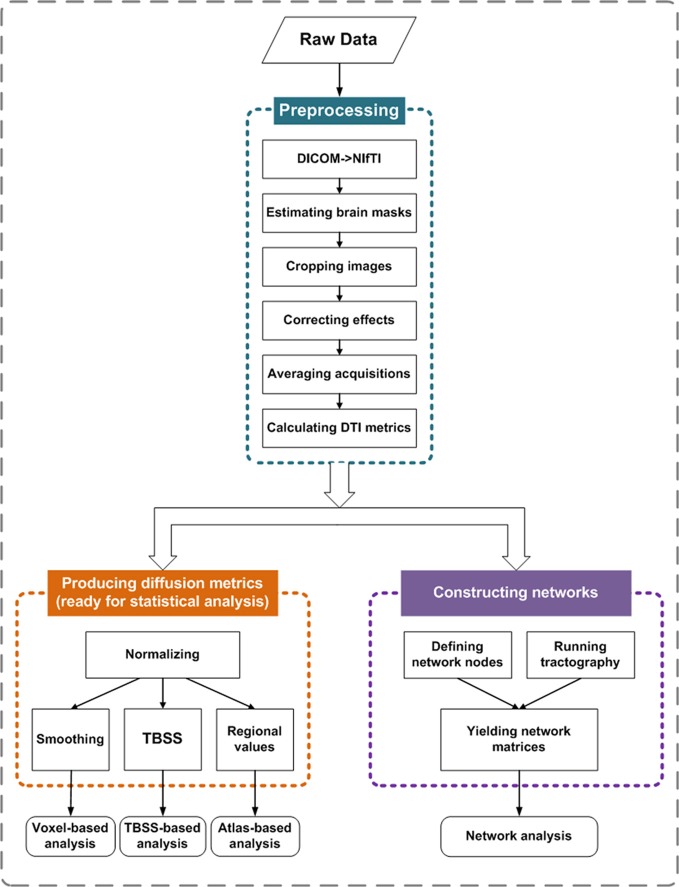
**Main procedure for pipeline processing of dMRI datasets in PANDA.** The procedure includes three parts: (1) preprocessing; (2) producing diffusion metrics that are ready for statistical analysis; and (3) constructing networks.

The main procedure of PANDA is shown in Figure 1 and includes three steps: (1) preprocessing; (2) producing diffusion metrics (ready for statistical analysis); and (3) constructing networks.

#### Preprocessing

***Converting DICOM files into NIfTI images.*** The input files of PANDA can be in either DICOM or NIfTI format. If the input files are in NIfTI format, this conversion step will be skipped. Otherwise, DICOM files will be converted into NIfTI format during this step. The *dcm2nii* tool embedded in MRIcron accomplished this task.

***Estimating the brain mask.*** This step yields the brain mask by using the *bet* command of FSL (Smith, 2002). The brain mask is required for the subsequent processing steps. Here, the b0 image without diffusion weighting was used for the estimation.

***Cropping the raw images.*** To reduce the memory cost and speed up the processing in subsequent steps we cut off the non-brain space in the raw images, leading to a reduced image size. The acquired brain mask was used to determine the borders of the brain along the three dimensions. The *fslroi* command of FSL was then applied to remove the non-brain spaces.

***Correcting for the eddy-current effect.*** Eddy-current induced distortion of diffusion weighted images (DWI), as well as simple head-motion during scanning, can be corrected by registering the DW images to the b0 image with an affine transformation. To achieve this, the *flirt* command of FSL was used. Notably, this registering procedure was applied to all images, with the b0 image of first acquisition used as the target if multiple DWI acquisitions were scanned. It is worth mentioning that while the *eddy_correct* command of FSL is not called here, the result of this step is exactly the same as the output of *eddy_correct*. Basically, PANDA just splits the 4D file (the input file of *eddy_correct*) into a number of 3D files and then performed the affine-registration exactly like *eddy_correct*. The purpose of this implementation is to avoid the large memory demand when the 4D file size is huge. Finally, the gradient direction of each DWI volume was rotated according to the resultant affine transformations (Leemans and Jones, 2009).

***Averaging multiple acquisitions.*** This step will be skipped if there is only one DWI acquisition. Otherwise, after eddy-current correction, the aligned multiple DWI was averaged by calling the *fslmaths* command of FSL.

***Calculating diffusion tensor (DT) metrics.*** This step involves a voxel-wise calculation of the tensor matrix and the DT metrics, including FA, MD, axial diffusivity (AD), and radial diffusivity (RD) (Pierpaoli and Basser, 1996; Song et al., 2002). The *dtifit* command of FSL was applied.

#### Producing diffusion metrics that are ready for statistical analysis

***Normalizing.*** To allow for comparison across subjects, location correspondence has to be established. To end this, registration of all the individual images to a standardized template is always applied. Here, PANDA non-linearly registered individual FA images of native space to the FA template in the MNI space by calling the *fnirt* command of FSL. The resultant warping transformations were then used to resample the images of the diffusion metrics (i.e., FA, MD, AD, and RD) into the MNI space with a customized spatial resolution (e.g., 1 × 1 × 1 mm or 2 × 2 × 2 mm). This resampling step was implemented by the *applywarp* command of FSL.

***Output for voxel-based analysis.*** The resultant images of the diffusion metrics in the standard space are ready for voxel-based statistical analysis. However, in the framework of voxel-based analysis, these images are typically smoothed to some degree, which can reduce the effect of image noise and misalignment between subjects. Accordingly, PANDA smoothed the images with a given Gaussian kernel, which was realized by calling the *fslmaths* command of FSL. The smoothed diffusion metric images can then be directly used for voxel-based statistical analysis with any preferred tools, e.g., FSL (http://www.fmrib.ox.ac.uk/fsl/), SPM (http://www.fil.ion.ucl.ac.uk/spm/), or AFNI (http://afni.nimh.nih.gov/afni/).

***Output for atlas-based analysis.*** In addition to the popular voxel-based method of analysis, diffusion metrics can be analyzed at the level of region of interests (ROI), which may provide better statistical sensitivity in some cases (Faria et al., 2010). Recently, a few WM atlases (e.g., the ICBM-DTI-81 WM labels atlas and the JHU WM tractography atlas) have been proposed (Mori et al., 2008). These WM atlases in the standard space allow for parcellation of the entire WM into multiple ROIs, each representing a labeled region in the atlas. To support ROI-based analysis, PANDA calculates the regional diffusion metrics (i.e., FA, MD, AD, and RD) by averaging the values within each region of the WM atlases. These resultant ROI-based data (saved as text files) can be statistically analyzed with SPSS (http://www-01.ibm.com/software/analytics/spss/) and other statistical packages.

***Output for TBSS-based analysis.*** The TBSS framework avoids the necessity of choosing a spatial smoothing procedure during voxel-based analysis and may provide better sensitivity and interpretability when it is applied to multi-subjects dMRI datasets (Smith et al., 2006). To support this type of analysis, PANDA follows the standard TBSS framework. Firstly, the mean of all the aligned FA images was created and skeletonized, resulting in a mean FA skeleton. Secondly, the diffusion metric data from individual subjects were projected onto the skeleton. Finally, individual images with data on the skeleton were created. The resultant images can be directly used for voxel-wise statistical analysis on the skeleton. Here, the *fslmaths* and *tbss_skeleton* commands of FSL were employed.

#### Constructing networks

**Figure 2:**
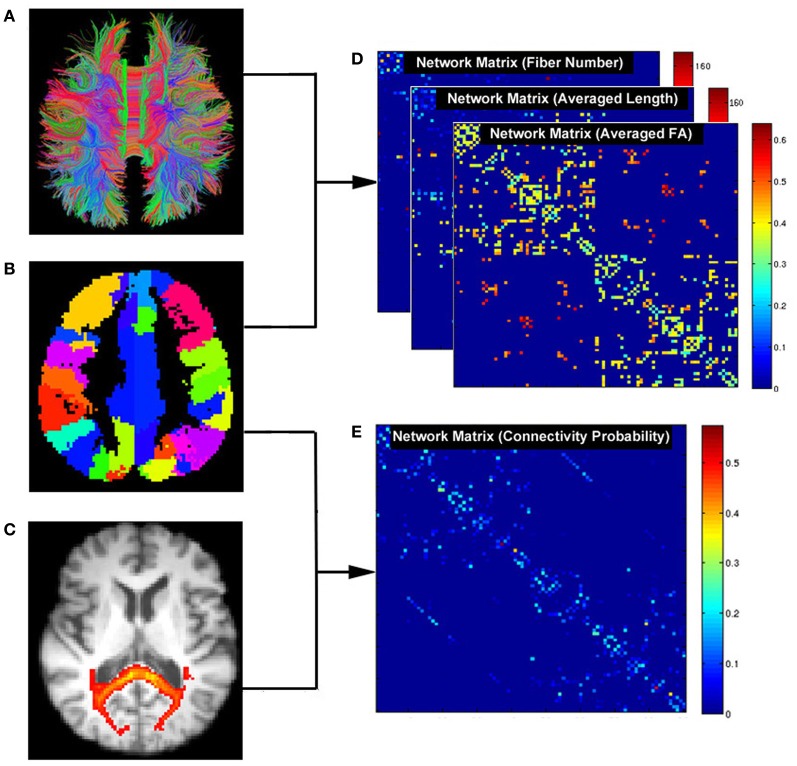
**Flowchart for constructing anatomical brain networks using diffusion tractography in PANDA. (A)** White matter tracts reconstructed using deterministic tractography. **(B)** Parcellation of gray matter in diffusion space. Each color represents a node in a brain network. **(C)** White matter connectivity maps using FSL probabilistic tractography. **(D)** Three resultant network matrices weighted by fiber number, averaged length, and averaged FA. **(E)** The network matrix weighted by connectivity probability.

Two basic elements are required for a network: a node and a connection. Thus, the central tasks for constructing brain networks are: (1) defining network nodes and (2) defining connections between nodes. The schematic flowchart of network construction is demonstrated in Figure 2.

***Defining network nodes.*** Typically, the entire brain is divided into multiple regions using a prior gray matter (GM) atlas, where each region represents a network node (Bullmore and Sporns, 2009). However, the prior atlases are generally defined in the standard space and need to be transformed to the native dMRI space of each individual. To address this, PANDA uses the framework proposed by Gong et al. (2009a). Specifically, the individual FA image in native space was co-registered to its corresponding structural image (i.e., T1-weighted) using an affine transformation. The individual structural image was then non-linearly registered to the ICBM152 template. Based on the resultant transformations in these two steps, an inverse warping transformation from the standard space to the native dMRI space can be obtained. Prior atlases in the standard space were then inversely warped back to individual native space by applying this inverse transformation. Currently, PANDA provides two well-defined atlases: the Automated Anatomical Labeling (AAL) (Tzourio-Mazoyer et al., 2002) atlas and the Harvard-Oxford atlas (HOA) (http://www.cma.mgh.harvard.edu/fsl_atlas.html). Notably, users can import customized atlases into PANDA to define the network nodes. During this step, the *flirt*, *fnirt*, *inwarp*, and *applywarp* commands of FSL were used.

*Constructing networks using deterministic tractography*. In general, deterministic tractography assumes a deterministic fiber orientation at every location during tracking, typically ending up with 3D trajectories for reconstructed WM tracts. Here, the *dti_recon* and *dti_tracker* commands of the Diffusion Toolkit (http://trackvis.org/dtk/) were called to reconstruct all possible fibers within the brain by seeding from all the WM voxels. For every pair of brain nodes/regions defined above, fibers with two end-points located in their respective masks were considered to link the two nodes. Based on the linking fibers, PANDA calculated three basic weighted matrices: *number-weighted matrix* (M*^N^*), *FA-weighted matrix* (M^FA^), and *length-weighted matrix* (M*^L^*). In the matrices, each row or column represents a brain region/node. The values of the elements M(*i, j*)*^N^*, M(*i, j*)^FA^, and M(*i, j*)*^L^* represent the number, averaged FA and averaged length of linking fibers between node *i* and node *j*, respectively. The resultant matrices were saved as a MATLAB data file and can be directly used for topological analysis with graph theoretic approaches (Bullmore and Sporns, 2009; Bullmore and Bassett, 2011).

*Constructing networks using probabilistic tractography*. In contrast, probabilistic tractography typically runs the tracking procedure many times, and fiber orientation is determined probabilistically. This type of tractography may improve tracking sensitivity, particularly for non-dominant fibers. The probabilistic tractography proposed by Behrens et al. (2003, 2007) has been implemented in FSL and is called by PANDA for network construction. This process involves two steps as follows:

*BedpostX*. Using the Markov Chain Monte Carlo sampling technique, this module estimated the local probability distribution of fiber direction at each voxel, a prerequisite for running subsequent probabilistic tractography (Behrens et al., 2003). In PANDA, bedpostX was realized by calling the *xfibres* command of FSL.

*Probabilistic Tractography and Network Construction*. Network construction using FSL-based probabilistic tractography has been previously described (Gong et al., 2009b). Briefly, for each defined brain region/node, probabilistic tractography was performed by seeding from all voxels of this region. For each voxel, 5000 fibers were sampled. To achieve this, the *probtrackx* command of FSL was called. The connectivity probability from the seed region *i* to another region *j* was defined by the number of fibers passing through region *j* divided by the total number of fibers sampled from region *i*. The connectivity probability of each node to the other nodes within the brain network can be calculated by repeating the tractography procedure for all nodes. This leads to an individual-specific weighted matrix, whose rows and columns represent the brain nodes and whose elements represent the connectivity probability between nodes. This matrix can also be directly used for various network analyses.

### Realization of pipelines

PSOM is a flexible framework for the implementation of pipelines in the form of Octave or Matlab scripts (Bellec et al., 2012), and was employed to build up the processing pipeline in our study. Here, a pipeline refers to a collection of jobs with a well identified set of options that use files for inputs and outputs. The entire processing flow of PANDA includes 41 steps, each of which is a job within the PANDA pipeline. Notably, more steps can be added if new functions or processing steps are included. The workflow of the current PANDA pipeline showing all the jobs and their associated dependencies is illustrated in Appendix A.

**Figure 3:**
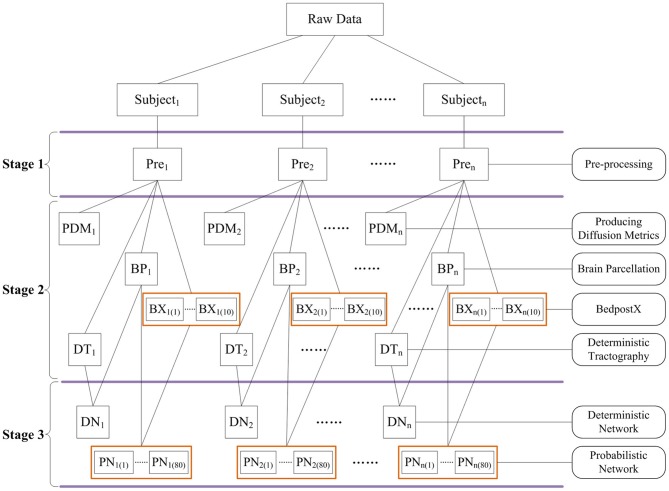
**The schematic parallelizing strategy of PANDA.** For example, pre-processing steps in Stage 1 are parallelizable across subjects. Independent processing steps from the same subject or across subjects in Stage 2 and Stage 3 can be parallelized as well. In addition, *BedpostX* and *Probabilistic Network Construction* have been internally parallelized, as indicated by orange boxes.

In particular, PANDA was designed to allow for jobs running in parallel either on a single computer with multiple cores or on a computing cluster. Notably, the PANDA processing steps are parallelizable at multiple levels. For example, the same processing steps (i.e., preprocessing) for a group of subjects can be parallelized, since the steps are independent across subjects. In addition, for the same subject, different processing steps without between-dependency such as *producing diffusion metrics* and *brain parcellation* can be parallelized as well. Finally, a few very time-consuming steps (i.e., *BedpostX* and *Probabilistic Tractography and Network Construction*) have been internally parallelized. The parallelizing strategies in PANDA are demonstrated in Figure 3.

### Testing the age effect on WM connectivity by using PANDA

#### Subjects

The test included data from 23 young adults (males, 11; females, 12; age, 17–24 years) and 17 elderly individuals (males, 8; females, 9; age, 54–77 years). All subjects were recruited from the campus and the local community. Subjects with a history of neurological or psychiatric disorders were excluded from this study. Written informed consent was obtained from each subject, and the protocol was approved by the Ethics Committee of the State Key Laboratory of Cognitive Neuroscience and Learning, Beijing Normal University.

#### MRI acquisition

All scans were performed using the 3-T Siemens Tim Trio MRI scanner in the Imaging Center for Brain Research, Beijing Normal University. Diffusion MRI was acquired using a single-shot echo-planar imaging-based sequence with following parameters: coverage of the whole brain; slice thickness, 2 mm; no gap; 68 axial slices; repetition time (TR), 9000 ms; echo time (TE), 92 ms; flip angle, 90°; 66 non-linear diffusion weighting directions with *b* = 1000 s/mm^2^ and one image without diffusion weighting (i.e., *b* = 0 s/mm^2^); 4 repetitive acquisitions; acquisition matrix, 128 × 124; field of view (FOV), 256 × 248 mm^2^; resolution, 2 × 2 × 2 mm. Three-dimensional T1-weighted images with high resolution were obtained using a three-dimensional magnetization prepared rapid gradient echo (MP-RAGE) sequence with the following parameters: 1 mm slice thickness without gap; 176 sagittal slices; TR, 1900 ms; TE, 3.44 ms; flip angle, 9°; acquisition matrix, 256 × 256; FOV, 256 × 256 mm^2^; resolution, 1 × 1 × 1 mm.

#### Image processing

The whole pipeline procedure of PANDA was run on all dMRI datasets with an in-house computing cluster of 6 nodes, each with 30GB of memory and 12 Intel Xeon E5649 2.53 GHz cores. For each pipeline step, default parameters were chosen.

#### Network topology

Graph theoretical approaches have been applied to characterize the topology of brain networks that are derived from neuroimaging data (Bullmore and Sporns, 2009). Here, we focus on two topological network parameters: global efficiency and local efficiency. Global efficiency was defined as the average of the inverse of the “harmonic mean” of the characteristic path length, which represents global information transferring ability within the network (Latora and Marchiori, 2001). Local efficiency quantifies the ability of the network fault tolerant, corresponding to the efficiency of the information flow between nodal neighbors. Specifically, local efficiency was defined as the average of nodal local efficiency that is computed as the global efficiency of the sub-graph composed by its nearest neighbors (Latora and Marchiori, 2001).

#### Statistical analysis

For diffusion metric, we tested the group difference on FA across the entire WM. Specifically, normalized and smoothed (6 mm Gaussian kernel) FA images produced by PANDA were employed for this voxel-based analysis. A general linear model (GLM) with gender being taken as a covariate was applied to each WM voxel. For multiple comparison correction, false discovery ratio (FDR) was applied, and *p* < 0.01 was considered as significant.

For each subject, the FA-weighted matrix generated from PANDA was selected for topological analysis. Each matrix is 78 × 78 and represents the WM network of cerebral cortex. Each row or column of the matrix represents a cortical region of the AAL template (Gong et al., 2009a,b). The global efficiency and local efficiency were then calculated. To test the group effect on the global and local efficiency, a GLM with gender and brain size as covariates was applied, and *p* < 0.05 was chosen as the significant level.

## Results

### An integrated matlab toolbox: PANDA

An integrated MATLAB toolbox named PANDA has been developed for fully automated processing of dMRI datasets, which is an open-source package and is freely available at http://www.nitrc.org/projects/panda. An online discussion forum (http://www.nitrc.org/forum/forum.php?forum_id=2731) and a mailing list (http://www.nitrc.org/mailman/listinfo/panda-commits) have been registered for PANDA, and technical supports and updates will be constantly provided by the developers. Notably, PANDA has been packaged with PSOM, MRIcron, and Diffusion Toolkit. Only FSL is required to be installed separately.

**Figure 4:**
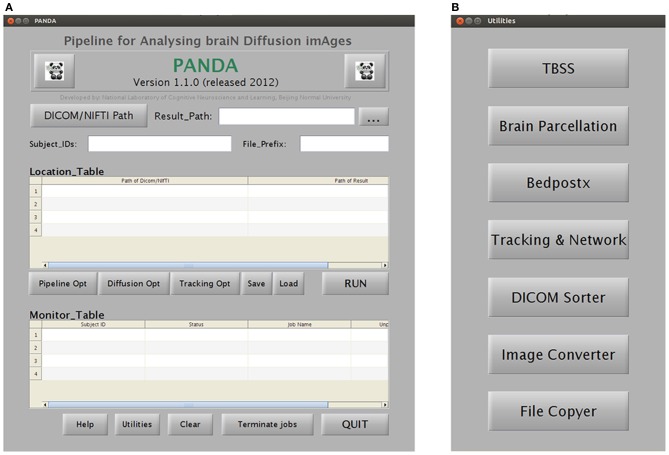
**A snapshot of the GUIs of PANDA. (A)** The main GUI for loading dataset and monitoring job status. **(B)** The GUI for initiating separate utilities.

Specifically, PANDA includes a main function and a set of separate modules/utilities. Using the main function, PANDA can run pipeline processing for any number of subjects, after raw dMRI datasets are loaded into the program. This running mode will finish all processing steps and end up with all outputs as described in “Materials and Methods.” In contrast, the utilities can be used separately for specific processing steps (e.g., DICOM conversion, TBSS, and brain parcellation). Particularly, PANDA has a very friendly GUI (Figure 4), with which users can perform various interactions with the embedded functions, e.g., setting inputs or outputs and configuring the processing parameters. In addition, PANDA can provide the status of the ongoing pipeline processing in real-time, allowing users to monitor progress through the GUI. The detailed descriptions for GUIs of PANDA are included in Appendix B.

As provided by PSOM (Bellec et al., 2012), PANDA has a number of advantages as follows: (1) it can run jobs in parallel either in a single computer with multiple cores or in a computing cluster; (2) it can generate log files and keep track of the pipeline execution; (3) if the program terminates before finishing, users can load a configuration file, click “RUN,” and PANDA will restart from the termination point; (4) if users re-run the pipelines after changing some options, PANDA will only restart the procedures related to these options; and (5) the jobs will run in the background and PANDA & MATLAB can be closed after clicking the “RUN” button.

### Resultant files of PANDA

**Figure 5:**
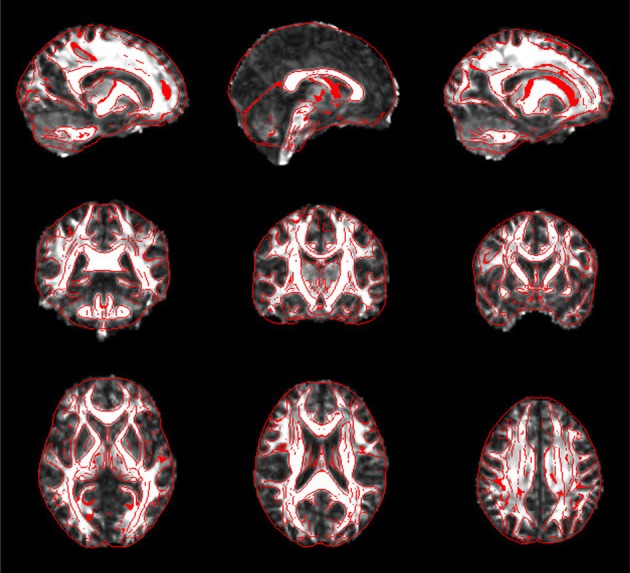
**Snapshot pictures for quality control of FA normalization.** The normalized FA is overlaid with image edges that were derived from the FA template. These pictures can be quickly viewed to check the quality of normalization.

For each subject, PANDA generates six folders containing resultant files, as listed in Table 1. Specifically, the *native_space* folder consists of all images and files in the native space. The files in the *quality_control* folder include 2D snapshot pictures of FA, T1, normalized FA, and normalized T1, which can be quickly viewed to check the quality of the data and related registrations (Figure 5). All files of the diffusion metrics that are ready for statistical analysis are stored in the folder named *standard_space*. The *trackvis* folder consists of resultant files generated by the “Diffusion Toolkit” for deterministic tractography, which can be opened with Trackvis. The *native_space.bedpostx* folder contains the resultant files of bedpostX that are required for FSL probabilistic tractography. Finally, the MATLAB files containing the network matrices with different weighting (i.e., fiber number, averaged FA, averaged length, and connectivity probability) are stored in the folder named *network*.

**Table 1:** **Folders produced by PANDA**.

**Folder name**	**Files**
*native_space*	Text files of bvals and bvecs
	Native-space images of DWI, b0, brain mask, FA, MD, AD, RD, and parcellation mask
*quality_control*	Snapshot pictures of native FA, native T1, normalized FA, and normalized T1
*standard_space*	Normalized images of FA, MD, AD, and RD (ready for voxel-based analysis)
	Text files of regional FA, MD, AD, and RD (ready for ROI-based analysis)
	Images of skeletonized FA, MD, AD, and RD (ready for TBSS analysis)
*trackvis*	Trackvis-related resultant files (for deterministic tractography)
*native_space bedpostx*	BedpostX-related resultant files (for probabilistic tractography)
*network*	MATLAB files containing network matrices weighted by fiber number, averaged FA, averaged length (from deterministic tractography), and connectivity probability (from probabilistic tractography)

### Time cost

To provide information about the time cost of PANDA procedures, a few baseline running-time tests were conducted. Specifically, two dMRI datasets with different acquisition schemes (dataset I: 64 directions, 4 repetitive acquisitions, resolution: 2 × 2 × 2 mm; dataset II: 30 directions, 2 repetitive acquisitions, resolution: 2.2 × 2.2 × 2.2 mm) were tested under four conditions (one subject with four cores; one subject with eight cores; two subjects with four cores; two subjects with eight cores). The results are listed in Table 2.

**Table 2:**
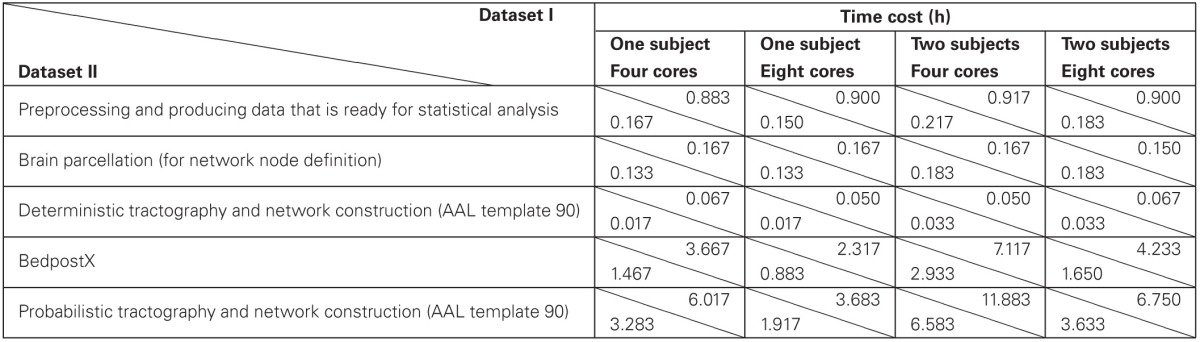
**Baseline time cost of pipeline processing on dataset I (64 DWI directions, 4 repetitive acquisitions, resolution: 2 × 2 × 2 mm) and dataset II (30 DWI directions, 2 repetitive acquisitions, resolution: 2.2 ×2.2 × 2.2 mm) with PANDA**.

*The processing was performed using a local workstation with 30 GB of memory and Intel Xeon E5649 2.53 GHz cores. Four conditions were tested: one subject with four cores; two subjects with four cores; one subject with eight cores; two subjects with eight cores*.

Obviously, the running time depends on dMRI scanning schemes. More DWI directions and more repetitive acquisitions will result in longer running time of *preprocessing* and *bedpostX*. Our results further demonstrated that the running-time for multiple subjects with multiple cores in PANDA can be effectively saved, due to the parallelized processing. For example, finishing the pre-processing steps for two subjects costs almost the same time as for one subject (Table 2). In addition, since the *bedpostX* has been parallelized internally, finishing *bedpostX* with eight cores cost only half of time as cost with four cores (Table 2).

### The age effect on WM connectivity using PANDA

**Figure 6:**
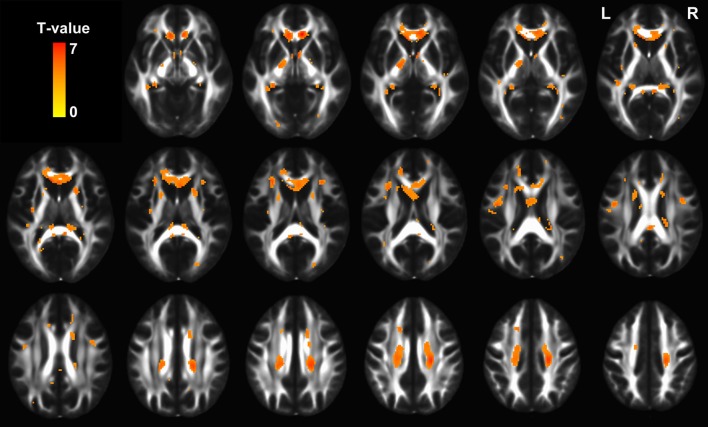
**The statistical map showing significant FA decreases in old group (*p* < 0.01, FDR corrected).** The hot color represents *t* values for the age effect.

As expected, voxel-based comparison revealed a distributed FA decreases (*p* < 0.01, FDR corrected) throughout the brain in the old group. Specifically, FA was mainly affected in the bilateral superior longitudinal fasciculus, uncinate fasciculus, internal capsules, external capsules, fornices, and corpus callosum (Figure 6).

**Figure 7:**
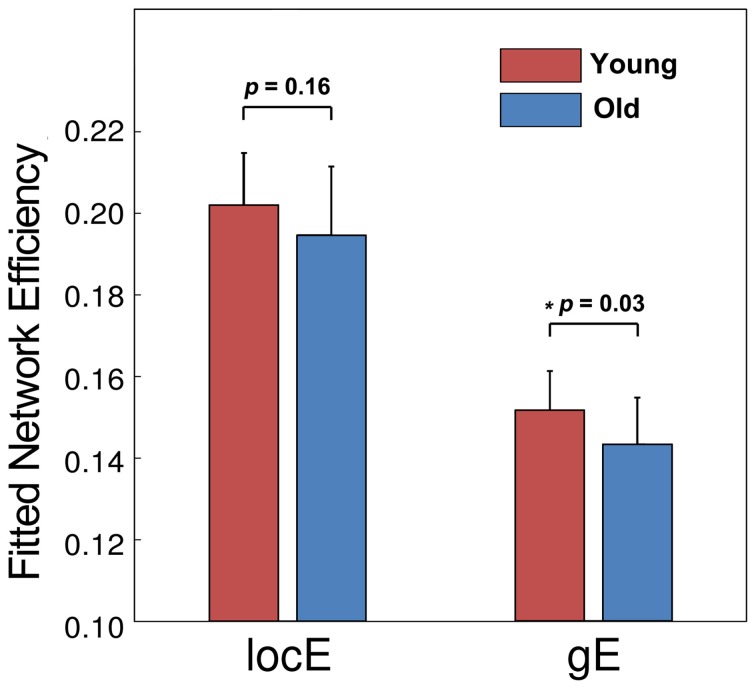
**The group comparison of network efficiency.** The old group showed a significant reduction of global efficiency and a trend of reduction in local efficiency.

Moreover, we observed group differences in topological efficiencies of WM network of cerebral cortex. As demonstrated in Figure 7, the global efficiency of the WM network showed a significant reduction in the old group (*p* = 0.03) after controlling for gender and brain size, and the local efficiency exhibited only a trend of reduction (*p* = 0.16).

## Discussion

In this study, we have developed a MATLAB toolbox named PANDA for comprehensively processing dMRI datasets. The key advantage of PANDA is that it fully automates all the processing steps of dMRI datasets for any number of subjects. PANDA can yield diffusion metric data that is ready for statistical analysis at three levels (voxel-level, atlas-level, and TBSS-level), and can generate anatomical networks/matrices of the entire brain using either deterministic or probabilistic diffusion tractography.

A fully automated pipeline naturally makes the data processing efficient, at the same time reducing potential mistakes by avoiding manual processing of individual steps. While constructing a dMRI processing pipeline with MIPAV (McAuliffe et al., 2001), JIST (Lucas et al., 2010), Nipype (Gorgolewski et al., 2011), or LONI (Dinov et al., 2009) is possible, it requires prior knowledge on pipeline design and programming skills related to these packages. In addition, knowledge on the details of all steps for processing dMRI dataset is required, which might be another challenge for end users. To provide a ready-for-use pipeline tool for end users, PANDA was developed, making it possible to process dMRI datasets immediately with established pipelines.

Notably, there exist differences in the processing procedures across existing dMRI packages, and some important processing steps might be overlooked (Jones et al., 2012). These issues have been well discussed by a few recent articles (Jones and Cercignani, 2010; Jones et al., 2012). The processing pipelines of PANDA have tried to follow the best practice as possible. For example, the adjustment of diffusion gradient directions after eddy-current correction, which has been frequently missed (Leemans and Jones, 2009; Jones et al., 2012), has been included in the PANDA pipeline. In future versions, PANDA will keep being updated to include processing steps of the best practice at the moment.

Another advantage of PANDA is that both sequential and parallel processing modes are supported, which makes it possible to take full advantage of available computing resources. The parallel environment can be either a single computer with multiple cores or a computing cluster, which increasingly enters into labs around the world. As shown in Figure 3, the PANDA processing have been parallelized as much as possible, and can thus reduce the time cost substantially under a parallel processing mode. For instance, the running time for pre-processing two subjects is almost the same as for one subject by using a workstation with four cores.

Finally, PANDA has a very friendly GUI (Figure 4), allowing the advanced users to select the desired options for each processing step. Depending on the datasets, users may change the options of some processing steps to optimize the processing quality. The reference data, e.g., image templates for normalization or prior atlases for node definition, can also be replaced by customized data, making it possible for processing dMRI data of non-human (e.g., primate) brains.

In the present study, we applied PANDA to produce results for testing the age effect on WM diffusion metrics as well as topological properties of the WM network. Significant FA reductions during aging were found in the bilateral uncinate fasciculus, superior longitudinal fasciculus, external capsules, fornices and corpus callosum, which are highly consistent with previous findings (Bennett et al., 2010; Michielse et al., 2010). In addition, significant reduction of global efficiency and a trend of reduction of local efficiency were observed in the old group. These topological changes are largely compatible with our previous results that are based on a larger dataset (Gong et al., 2009b). The declined WM connectivity and topology may underlie various patterns of cognitive decline during normal aging. The results for this specific study prove the usability and validity of the PANDA processing.

PANDA is of great applicability in the area of connectivity neuroscience. For example, this tool can be applied to dMRI datasets that are collected to study various connectivity hypotheses. Also, the effects of dMRI processing parameters or steps on the final connectivity results can be easily tested by using PANDA. Recently, the term “connectome” has been proposed to advocate efforts for comprehensively mapping and analyzing brain connectivity and networks (Sporns et al., 2005), and dMRI has been taken as a primary technique for structural macro-connectome (Behrens and Sporns, 2012). This will lead to a large number of dMRI datasets in the foreseeable future (http://humanconnectome.org/). To process these connectome dataset, PANDA has unique advantages, as it can handle the large number of datasets very efficiently because of its parallelizing strategies. Meanwhile, it can automatically provide important metrics of interest (e.g., diffusion metrics of brain connectivity and brain network matrices) for connectome studies. Therefore, PANDA can potentially make contributions to the study of the human connectome in the near future.

In summary, PANDA can substantially facilitate/simplify image processing in a dMRI-related study, and can provide measures for WM connectivity and network analysis. It has an extendable design framework, and new functions or utilities can and will be added in the future.

### Conflict of interest statement

The authors declare that the research was conducted in the absence of any commercial or financial relationships that could be construed as a potential conflict of interest.

## Figures and Tables

**Figure A1:**
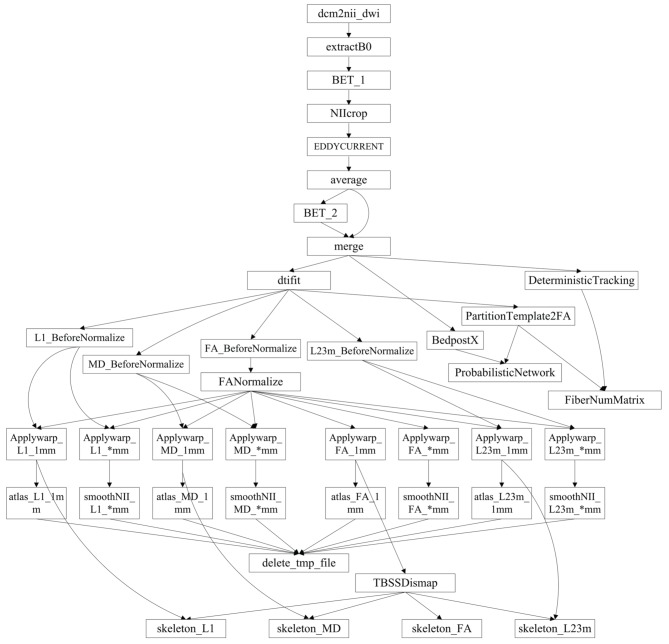
**Implementation of the PANDA pipeline.** The entire process of the PANDA pipeline was divided into 41 steps. Arrows indicate dependencies: A→B means that B cannot start until A is finished.

**Figure B1:**
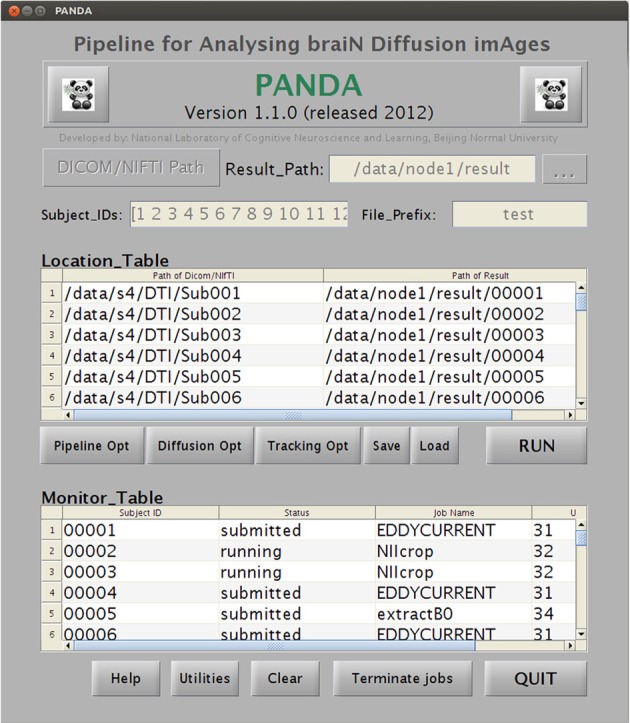
**A snapshot of the main GUI while PANDA is running.** The GUI allows for (1) inputting raw dMRI datasets and configuring processing parameters and (2) monitoring the progress of data processing in real-time.

**Figure B2:**
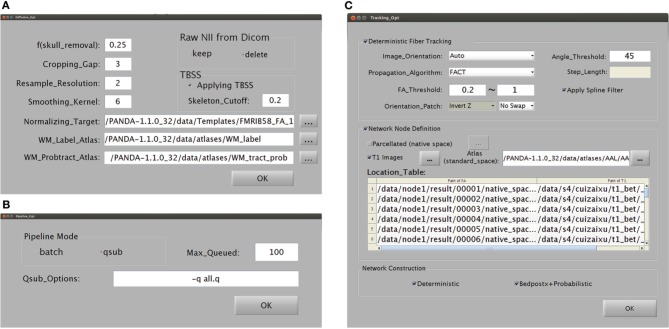
**Snapshots of the GUI for configuring the processing parameters. (A)** A snapshot of the GUI for changing the preprocessing parameters and for producing diffusion metrics that are ready for statistical analysis. **(B)** A snapshot of the GUI for changing the computing modes of PANDA. **(C)** A snapshot of the GUI for changing the parameters used to construct anatomical brain networks.

**Figure B3:**
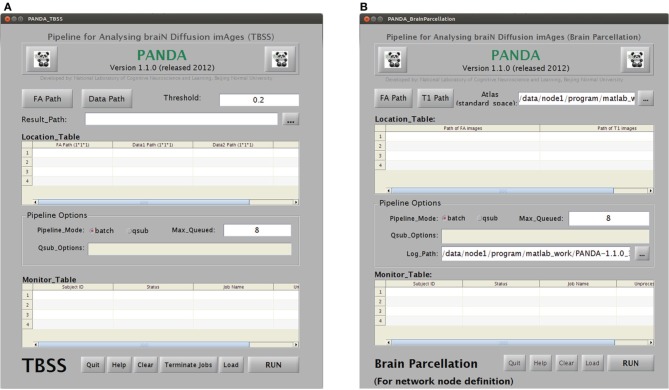
**GUIs for the utilities “TBSS” and “brain parcellation” in PANDA. (A)** The utility for TBSS analysis. **(B)** The utility for brain parcellation.

**Figure B4:**
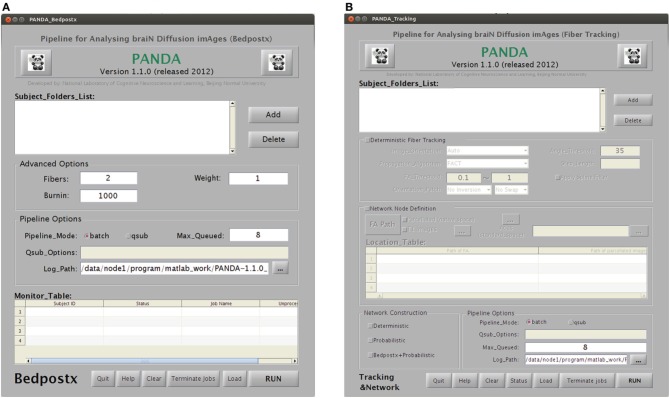
**GUIs for the utilities “Bedpostx” and “Tracking & Network” in PANDA. (A)** The utility for Bedpostx. **(B)** The utility for tractography and network construction.

**Figure B5:**
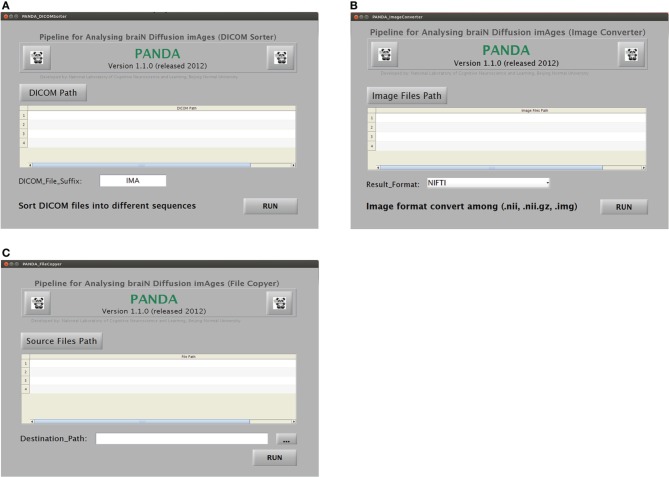
**GUIs for the utilities “DICOM Sorter,” “Image Converter,” and “File Copier” in PANDA. (A)** The utility for DICOM Sorter. **(B)** The utility for Image Converter. **(C)** The utility for File Copier.

## Appendices

### Appendix B: GUIs of PANDA

#### Main function

The main GUI of PANDA is shown in Figure B1. Users are required to set up inputs and configure outputs through this GUI. Specifically, the data inputs are folders, each containing files in either DICOM or NIfTI format, for each subject. The output configuration includes: (1) a main output folder that contains subject-specific subfolders of results; (2) digital subject IDs; and (3) a prefix. The IDs and prefixes are used to name the resultant subfolder or files for each subject. In addition, users may change the pipeline options (Figure B2A), diffusion options (Figure B2B), and tracking options (Figure B2C). The default setting for these options will be used if no changes are made.

Once all required settings are established, users simply click the “RUN” button to start the processing. PANDA will automatically finish all the sequential jobs and yield files containing diffusion metrics and anatomical brain networks, as described in the “Materials and Methods.” During processing, the status of jobs can be checked in the monitor table of the GUI (Figure B1).

#### Separate utilities

***TBSS.*** As shown in Figure B3A, this utility is for separate TBSS procedures, which require all images of FA and other diffusion metrics to be aligned in the MNI space. With correct input settings, this module will automatically generate individual images with data on the skeleton for all subjects. Statistical analyses can be directly applied to the resultant images.

***Brain parcellation (node definition).*** This utility is used to separately define the brain network nodes. The sub-GUI is shown in Figure B3B. This module requires FA images of native space and skull-stripped T1 images as inputs. A prior atlas in the MNI space should also be specified. The results of this utility are individual atlas images in the dMRI native space for all subjects. These images can be directly loaded by the utility “Tracking & Network.”

***Bedpostx.*** As shown in Figure B4A, this utility allows for the estimation of voxel-wise local probability distributions of fiber orientation for a set of subjects, which is typically very time-consuming. The input for each subject should be a folder containing four files as listed: (1) a 4D image named *data.nii.gz* containing diffusion-weighted volumes and volumes without diffusion weighting; (2) a 3D binary brain mask volume named *nodif_brain_mask.nii.gz*; (3) a text file named *bvecs* containing gradient directions for diffusion weighted volumes; and (4) a text file named *bvals* containing the b-values that were applied to each volume acquisition. This module will generate a separate folder containing all the files that are required for subsequent probabilistic tractography.

***Tracking & Network.*** This utility can separately construct anatomical brain networks based on tractography. The sub-GUI is shown in Figure B4B. For a deterministic tractography-based network, a folder with four files described in the section “Bedpostx” together with an individual-specific atlas image generated by the utility “Brain Parcellation” are required. For a probabilistic tractography-based network, the resultant folder of the utility “Bedpostx” and the individual-specific atlas image should be the inputs. As described in the “Materials and Methods,” this module will generate network matrices that are saved in a MATLAB data file.

***DICOM sorter.*** This handy utility, as shown in Figure B5A, can automatically sort multiple DICOM files in the same folder into sequence-specific or subject-specific sub-folders, based on the header information of the DICOM files. This is particularly useful when the DICOM files from different sequences or subjects are saved in the same folder, which happens very often.

***Image converter.*** The NIfTI format can be a pair of files (hdr/img), a single file (nii), or a compressed file (nii.gz). A NIfTI file may be required in a certain file type, e.g., ^*^.nii or ^*^.hdr/img. As shown in Figure B5B, this utility can convert NIfTI pair format (hdr/img), NIfTI format (nii), and NIfTI GZ format (nii.gz) file types.

***File copier.*** This utility can copy a large number of files located in different source folders into the same target folder. The sub-GUI is shown in Figure B5C. After PANDA processing, each subject will have unique folders containing the resultant files. “File Copier” can easily copy the same types of resultant files (e.g., aligned FA images) of all the subjects to one target folder, which might be helpful for further statistical analysis or other purposes.
